# Supplementary figures and images for: Genome-wide characterization and expression analysis of the JRL gene family in response to hormones and abiotic stress in tomato (Solanum lycopersicum L.)
Source: PeerJ. 2025 Jul 21;13:e19724. doi: 10.7717/peerj.19724 (PMC12288746; doi:10.7717/peerj.19724)

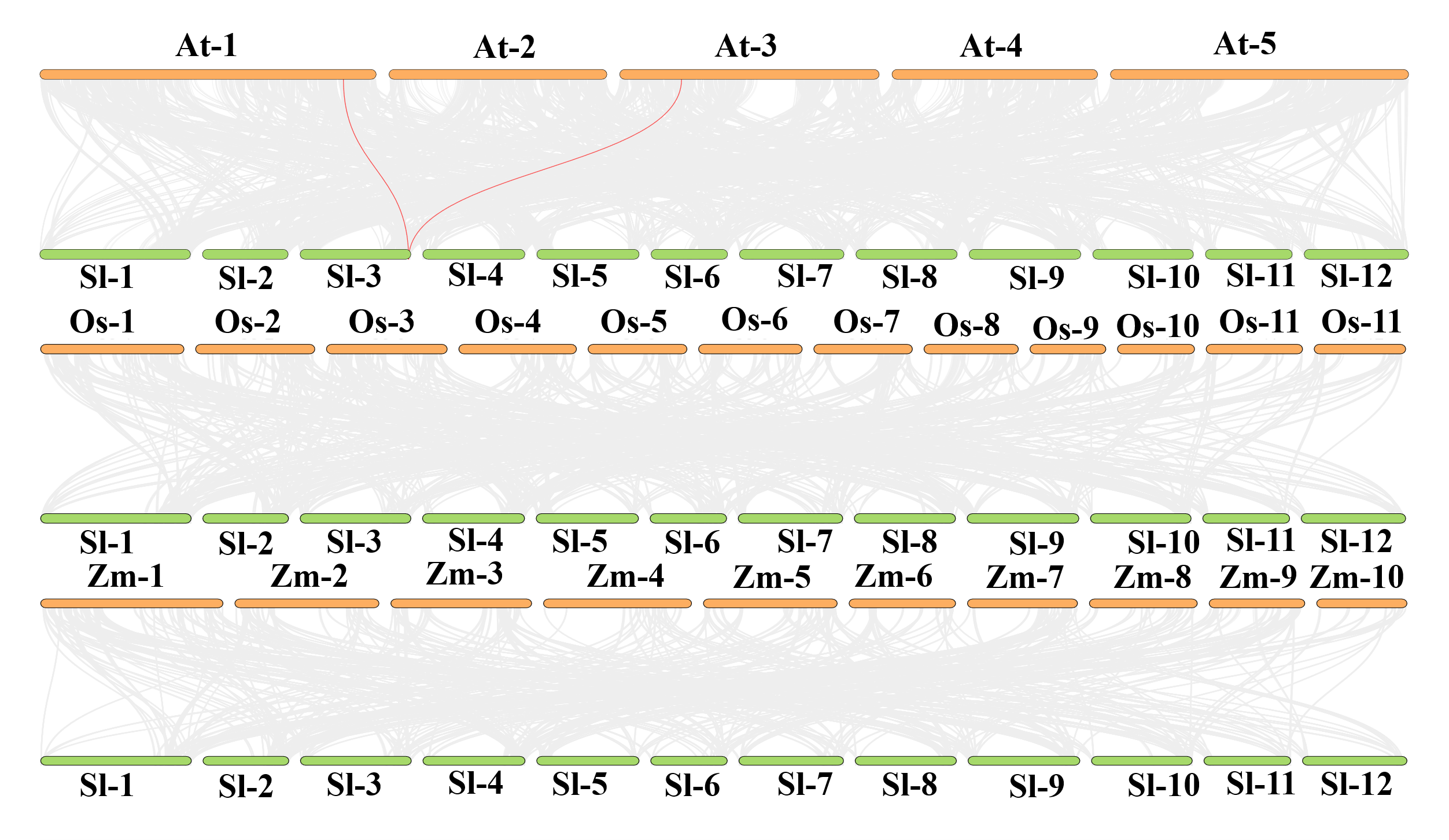

Supplement: Supplemental Information 1 — The gray lines represent collinearity in the wide genome of tomato and other plants, while the red lines highlight the collinear SlJRL gene pairs. [file peerj-13-19724-s001.png]

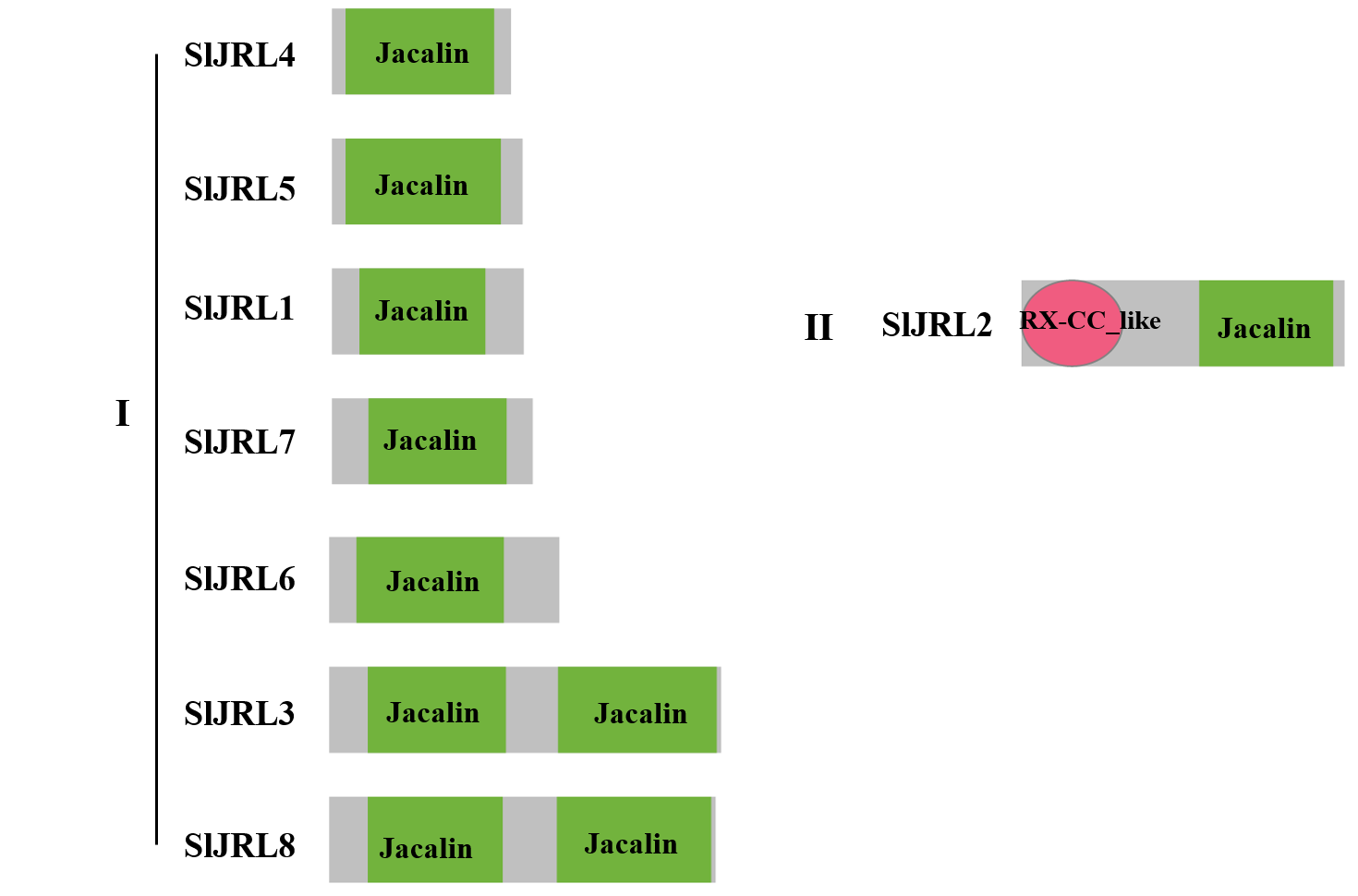

Supplement: Supplemental Information 2 — Two types of structural domains exist for all proteins: type I and type II. [file peerj-13-19724-s002.png]
