# Supplementary material for: Genome-wide characterization and expression analysis of the JRL gene family in response to hormones and abiotic stress in tomato (Solanum lycopersicum L.)
Source: PeerJ. 2025 Jul 21;13:e19724. doi: 10.7717/peerj.19724 (PMC12288746; doi:10.7717/peerj.19724)
Supplement: Supplemental Information 6 [file peerj-13-19724-s006.docx]

| Table S4 Conserved motifs in the amino acid sequence of SlJRL proteins | | |
| --- | --- | --- |
| Motif | Width Multi level | Consensus sequence |
| 1 | 17 | JRSIKFYTNKGKYGPFG |
| 2 | 29 | KHGGDGGENLKAIILDYPSEYLTGISGSY |
| 3 | 30 | MIKVGAWGGSGGSEWEEKGKGPVKEIFIAY |
| 4 | 29 | TYFSSSMAGGKIVGFHGKSGLFLDAIGVH |
| 5 | 38 | PDKMDVMKGIQPRCPGPWGGCSGKGWDDGVFCTIKQVQ |
| 6 | 19 | THFNFVIKNHGLFGGFHGT |
| 7 | 15 | SLQFLYYEDGNFVQS |
| 8 | 7 | SFWSQLH |
| 9 | 14 | SDSALEAIGIYVKP |
| 10 | 15 | NLVVKSLCFVTNAKC |
